# The Impact of Nutrition on Endometriosis Complaints in Patients Using and Not Using Hormone Therapy

**DOI:** 10.3390/nu17172889

**Published:** 2025-09-06

**Authors:** Agnieszka Pelc, Ewelina Polak-Szczybyło

**Affiliations:** 1 Department of Physiotherapy, Institute of Health Sciences and Psychology, Collegium Medicum, University of Rzeszów, 35-959 Rzeszow, Poland; agpelc@ur.edu.pl; 2 Department of Dietetics, Faculty of Health Sciences and Psychology, Collegium Medicum, University of Rzeszów, ul. Warzywna 1a, 35-959 Rzeszow, Poland

**Keywords:** endometriosis, nutrition, hormone therapy, menstrual symptoms, menstrual pain

## Abstract

Background: Endometriosis is a chronic inflammatory disease marked by the presence of endometrial tissue outside the uterus. Main symptoms include pain in the sacrum, pelvis, and abdomen, occurring at various stages of the menstrual cycle or during intercourse. These symptoms can severely affect daily functioning and quality of life. Methods: The study involved 200 women aged 18–47, divided into two groups. The WHT group (N = 100) included women with endometriosis not receiving hormone therapy, and the HT group (N = 100) included women undergoing hormone treatment. An anonymous questionnaire was used, comprising a VAS, the FFQ-6 food frequency questionnaire, and questions regarding menstruation-related symptoms and effects. Results: Women in the HT group reported higher pain levels (Me = 8.0 vs. 7.0) and more frequent negative impacts on academic/work performance (*p* = 0.008) than the WHT group, who reported higher work attendance (*p* = 0.043). In the WHT group, consumption of sugar, honey (*p* = 0.019), sweet cereals (*p* = 0.023), and sweetened beverages (*p* = 0.036) was associated with absences and concentration difficulties (*p* = 0.010). In contrast, in the HT group, those reporting absences consumed more nuts and vegetables (*p* = 0.024; *p* = 0.003). Conclusions: Women with endometriosis undergoing hormone therapy report more severe pain and more frequent disruptions in daily functioning. Both hormone therapy and diet significantly influence the intensity of menstrual symptoms as well as the ability to function professionally and socially.

## 1. Introduction

Endometriosis is a chronic inflammatory disease characterized by the presence of endometrium outside the uterus [1]. Despite many theories explaining its pathogenesis, its etiology remains unknown. Its development and progression are influenced by various factors, including genetic, immunological, environmental, and hormonal [2]. It is estimated that it affects 10–15% of women of reproductive age, with the highest incidence between 25 and 35 years of age. This means about 190 million women worldwide and up to 1 million cases in Poland [3,4,5]. Inflammation plays an important role in the pathogenesis of endometriosis and is the main cause of pain associated with the disease. Pro-inflammatory cytokines, such as interleukin-1, interleukin-6, tumor necrosis factor alpha and prostaglandins, play a role in these processes. Their excessive amount and activity can lead to increased inflammation, exacerbating symptoms associated with endometriosis. These substances can also stimulate each other’s production and generate a new inflammatory environment [6]. The main symptoms of endometriosis include pain in the sacral, pelvic, and abdominal areas, which may occur at various stages of the menstrual cycle or even during sexual intercourse. These symptoms can severely impact daily functioning and quality of life [7,8]. Additional symptoms may include heavy menstrual bleeding, chronic fatigue, painful bowel movements (dyschezia), painful urination (dysuria), hematuria, constipation, and infertility [9].

As many of these symptoms are directly linked to the menstrual cycle, it is essential to understand its typical characteristics. Menstruation typically begins at puberty and occurs regularly every 24 to 38 days, with bleeding lasting 5 to 8 days [10]. Many women experience menstrual-related issues, such as dysmenorrhea, which is defined as cyclical pain in the lower abdomen or pelvis that can radiate to the lower back, legs, and inner thighs [11]. The pain typically lasts between 8 and 72 h and may be accompanied by symptoms such as headaches, dizziness, irritability, diarrhea, nausea, vomiting, mood changes, and sleep disturbances [12,13]. Studies estimate that between 45% and 95% of women suffer from dysmenorrhea [11,14]. When this problem has no organic basis and is not the result of any pathological changes, it is called primary dysmenorrhea [12]. Secondary dysmenorrhea is menstrual discomfort that occurs due to pelvic disorders or is the result of an existing medical diagnosis. The most common cause is endometriosis [15]. One of the methods of dealing with painful periods is hormone therapy. Hormonal therapies alleviate the symptoms of dysmenorrhea by thinning the endometrial lining and reducing the production of cyclooxygenase-2 and prostaglandins [16]. Commonly reported side effects of oral contraceptives vary depending on the type and formulation. For example, combined oral contraceptives are often associated with nausea, headaches, breast tenderness, and weight gain, while progestin-only pills are more frequently linked to irregular bleeding and spotting [17].

Studies on the relationship between dietary factors and endometriosis are gaining increasing interest, as it has been noted that diet can affect physiological and pathological processes associated with this disease [18,19]. To date, no studies have specifically focused on the analysis of dietary patterns among women suffering from endometriosis and dysmenorrhea. Our study complements existing knowledge by also considering the impact of hormonal treatment. The aim of this study is to assess the impact of dietary habits and patterns on the severity of pain and associated symptoms during menstruation in women living in Poland who have been diagnosed with endometriosis, including those undergoing hormone therapy and a control group.

## 2. Materials and Methods

### 2.1. Study Participants

The observation group consisted of a total of 200 subjects. One group (N = 100) consisted of women with endometriosis without hormone therapy (WHT), while the other group (N = 100) consisted of women with hormone therapy (HT). The above division formed the basis of statistical analysis. Anthropometric characteristics were compared between subgroups: age, weight, height and BMI. In all of the above, no statistically significant differences were observed between the WHT group and the HT group, with mean scores for all subjects of 31.5 years, 65.3 kg, 167.0 cm, and 23.4, respectively (Table 1). All participants in the HT group used various contraceptives.

### 2.2. Study Qualification

Inclusion criteria were as follows: age 18–50 years, diagnosed endometriosis, no diagnosed comorbidities, no previous abdominal surgery and informed consent to participate in the study. Exclusion criteria for both groups were as follows: age < 18 and >50 years, no diagnosed endometriosis, diagnosed comorbidities, history of abdominal surgery, and no informed consent to participate in the study. The study was divided into 3 stages. In the first stage, 300 women with endometriosis were invited to participate in the study. In the second stage, the women completed an anonymous questionnaire about the quality of their nutrition and the symptoms and effects of menstruation. In the third stage, 100 women undergoing hormonal treatment (HT) were selected after taking into account inclusion and exclusion criteria. Women without hormone treatment (WHT) were matched to these women in terms of age, height, and weight.

The minimum study group was calculated based on the sample size calculator [20,21]. The data for estimating the minimum sample size was calculated based on the number of 100,000 women with endometriosis in Poland [4]. The statistical power of our study was 0.84, the recommended minimum power was 0.8 and the required number of subjects was 197.

### 2.3. Questionnaire

The survey questionnaire contained 26 questions. The first part of the survey included questions about age, place of residence, education, and the occurrence of possible diseases. The second part of the questionnaire included questions about the age at the onset of the first menstruation, the average cycle length, the intensity of menstrual bleeding, the symptoms accompanying the menstruation, and the intensity of pain experienced by the surveyed women during the last 6 months. Pain intensity was assessed using a visual analog scale (VAS). The visual analogue scale is typically a 10 cm line labelled with descriptors at the ends, such as “no pain” and “worst imaginable pain.” Subjects indicate the magnitude of pain by marking the line. A ruler is used to quantify the measurement on a scale of 0 to 10 cm [22]. The last part of the survey consisted of questions based on the FFQ-6 questionnaire. The FFQ-6 questionnaire was used for this scale, for this purpose, and the intake consumption of 62 food groups during the past 6 months was assessed using this scale. When responding to indicate the frequency of consumption, the respondents could choose one of six categories: 1—never or almost never; 2—once a month or less often; 3—several times a month; 4—several times a week; 5—every day; 6—several times a day [23]. An anonymous survey is attached as Supplementary File S1.

### 2.4. Statistical Analysis

Statistical analysis of the material was developed in Statistica 13.3 (StatSoft, Kraków, Poland) and the database and graphic design in Microsoft Word and Microsoft Excel. Descriptive statistics were calculated, specifying the mean and standard deviations, median values and quartiles (upper and lower), minimum and maximum values, and the number was given. The non-parametric Mann–Whitney U test was used to assess the significance of differences between the study and HT groups due to the failure to meet the assumptions of the parametric test (lack of compliance of the variable distribution with the normal distribution verified by the Shapiro–Wilk W test or the ordinal measurement scale). In the case of nominal data, Chi-square independence tests were used, and the examined relationships with data placed on the ordinal scale were performed using the Spearman rank correlation coefficient. The scale defining the strength of the relationship was adopted to describe and interpret the significant correlation as follows:

rxy = 0—no correlation

0 < rxy < 0.1—slight correlation;

0.1 < rxy < 0.3—weak correlation;

0.3 < rxy < 0.5—average correlation;

0.5 < rxy < 0.7—high correlation;

0.7 < rxy < 0.9—very high correlation;

0.9 < rxy < 1—almost full correlation.

The level of statistical significance was considered to be *p* < 0.05.

### 2.5. Ethics

In accordance with Polish national regulations, particularly the Act of 5 December 1996 on the Professions of Physicians and Dentists (Journal of Laws 2023, item 1516, as amended), the present study did not constitute a medical experiment and therefore did not require approval from a bioethics committee. Participation in the study was entirely voluntary, anonymous, and involved no interventions in the physical or psychological integrity of the participants. All respondents were adults who provided informed consent prior to participation. The purpose of the study was clearly stated in the introduction to the questionnaire, and no personal or identifying data were collected.

## 3. Results

### 3.1. Characteristics of the Menstrual Cycle and Menstrual Complaints in Both Groups

The significance of the differences between the WHT and HT groups was examined with reference to the age of menarche (in years), the occurrence of pain before menstruation (in days), the average length of the menstrual cycle (in days), the average bleeding time (in days), the average level of pain experienced during menstruation (scale 0–10), and the self-assessment of the intensity of bleeding during menstruation and the discomfort of menstruation. The only statistically significant difference between the subgroups was observed for the average level of pain experienced during menstruation. Women in the HT group had significantly higher levels of perceived pain compared to women in the WHT group (Me = 8.0 vs. 7.0) (Table 2).

Correlations between symptoms accompanying menstruation (absence from work/school, lack of concentration, reduced physical activity, adverse effects on academic/professional performance, elevated body temperature, worse mood) and subgroups were determined. Respondents in the WHT group were significantly more likely to be absent from work (N = 68) than participants in the HT group; when it came to adverse effects on academic/professional performance, women in the HT group were more likely to indicate the above problem (N = 79) (Table 3).

### 3.2. The Influence of Product Consumption on Menstrual Symptoms in the WHT Group

Statistical analysis of the relationship between frequency of food consumption and duration of symptoms during menstruation showed that in the WHT group, cookies were consumed less frequently in the case of more severe abdominal cramps (R = −0.20), red meat in the case of more frequent abdominal pain (R = −0.30), weakness (R = −0.20) and abdominal cramps (R = −0.26), as well as cold meats, frankfurters, sausages, and kabanos sausages in the case of more severe abdominal pain (R = −0.20). Positive significant correlations were observed between salty snacks and nausea/vomiting (R = 0.22), natural milk and backache (R = 0.22) and headache (R = 0.22), corn or sweet, flavored breakfast flakes and headache (R = 0.20), weakness (R = 0.20), nausea/vomiting (R = 0.21), buckwheat, barley, brown rice, wholemeal pasta and irritability/anxiety (R = 0.21), and between vegetable fats and lower abdominal pain (R = 0.29), weakness (R = 0.25), cramps felt in the lower abdomen (R = 0.21), irritability/anxiety (R = 0.22).

The next stage of statistical analysis was related to examining the differences in the frequency of food product consumption due to absence from work/college, lack of concentration, limitation of physical activity, adverse effect on academic/professional performance, increased body temperature, or worse well-being individually in the WHT group and HT group. The tables below (Table 4 and Table 5) include only statistically significant relationships. In the WHT group, taking into account the frequency of consumption and absence from work or college, the following were noted for the consumption of sugar and honey (*p* = 0.019), corn or sweet, flavored breakfast flakes (*p* = 0.023), and sweetened flavored drinks (*p* = 0.036). Subjects who had concentration problems reached for the above products significantly more often (*p* = 0.010). A relationship was also observed between people who limited their activity for the consumption of juices, nectars, and drinks. Subjects without activity restrictions consumed the above products significantly more often (*p* = 0.045). In a given group, with an unfavourable effect on the results at work/study, there is a lower consumption of sugar, honey (*p* = 0.045), corn or sweet, flavored breakfast flakes (*p* = 0.006), groats, white rice, white pasta (*p* = 0.009), vegetable fats (*p* = 0.036), juices, nectar, beverages (*p* = 0.017), and fast food (*p* = 0.037). Statistically significant relationships were also observed for the frequency of consumption of corn or sweet, flavored breakfast flakes and sugar/honey. Respondents who indicated an increase in body temperature consumed flakes more often compared to women who did not experience these symptoms (*p* = 0.036), while women who felt worse were significantly more likely to consume sugar and honey (*p* = 0.015) (Table 4).

### 3.3. The Influence of Product Consumption on Menstrual Symptoms in the HT Group

In the HT group, it was observed that with increased consumption of the given products, there was a more frequent occurrence of tenderness (for the consumption of salty snacks (R = 0.23) and sweetened carbonated beverages (R = 0.20), nausea/vomiting with white bread (R = 0.21), back pain and cramps felt in the lower abdomen with animal fats (R = 0.23; R = 0.20). With increasing symptoms, oat, barley, and rye flakes were consumed less frequently in the case of lower abdominal pain (R = −0.27), dark bread (R = −0.25), cornflakes or sweet, flavored breakfast cereals (R = −0.22), and fish (R = −0.22) in the case of back pain (R = −0.22), and fish (R = −0.21). In case of weakness, the consumption of dark bread (R = −0.22), fish (R = −0.21) and oat, barley, and rye flakes during abdominal cramps (R = −0.22), eggs and egg dishes (R = −0.21), and fish (R = −0.20) during irritability/nervousness, as well as fresh fruit (R = −0.20), poultry (R = −0.22), and fish (R = −0.23) during breast tenderness. Detailed data on the correlation between frequency of product consumption and symptoms are presented in Supplementary Table S1.

Taking into account the frequency of consumption of products and absence from work/college, it was observed that in the HT group who gave affirmative responses consumed more nuts and vegetables compared to the rest (*p* = 0.024; *p* = 0.003). Women who had problems with concentration due to menstruation consumed yellow, processed, and blue cheese significantly less frequently (*p* = 0.047), and reached for oat, barley, and rye flakes more frequently (*p* = 0.033) in contrast to those who did not have this problem. In terms of differentiation of the diet depending on physical activity, it was observed that women practicing physical activity without restrictions significantly more frequently consumed white bread (*p* = 0.017), and less frequently consumed oat, barley, and rye flakes (*p* = 0.030), buckwheat, barley, brown rice, whole-meal pasta (*p* = 0.009), and groats, white rice, white pasta (*p* = 0.024). The frequency of food consumption was then differentiated due to the adverse effect on academic/professional performance due to menstruation. In women who did not observe the above symptoms, a higher consumption of animal fat was noted (*p* = 0.025) (Table 5).

## 4. Discussion

Painful menstruation is the most common cause of absence from work or school [24]. In the present study, this applied to approximately two-thirds of respondents. Other research indicates that 20–40% of women miss school classes or are forced to take a break due to dysmenorrhea, and 40% of them experience a negative impact on academic performance and concentration [25]. In our study, most respondents reported problems with concentration, and one-third experienced adverse effects on their work or school results. In an analysis by Grandi et al. [26] including over 400 patients with dysmenorrhea, moderate pain was reported by 47% of participants, while severe pain, according to the VAS, was reported by 17%. In the present study, the average pain intensity, regardless of the group, was assessed at 7/10. Moreover, women with HT more frequently reported greater menstrual pain (7.5 in VAS) than woman WHT (6.8 in VAS). In a study of women with endometriosis who chose hormonal therapy, menstrual pain was observed at an average level of 9.61 on the same scale used in our study [27]. After one cycle of hormonal therapy, the VAS score decreased to approximately 5.0, and after three cycles to about 3.5–4.0 [28]. In contrast, among women without HT, moderate pain was most commonly recorded at VAS scores of 5 to 6, while severe pain ranged from 8 to 9 [26]. The results of our research may be related to the methods used in the treatment of dysmenorrhea, which include both nonsteroidal anti-inflammatory drugs (NSAIDs) [29] and hormonal birth control [30]. Hormonal contraception has been shown to reduce pain associated with dysmenorrhea [31]. Therefore, it may represent one of the first therapeutic choices for physicians in managing painful menstruation in endometriosis. In a study by Vercellini et al. [32], combined oral contraceptives were found to be effective not only in reducing recurrence after surgical removal of endometrial lesions but also in alleviating pain associated with this condition [33]. Sesti et al. [34] investigated the combined effect of hormone therapy and dietary modifications in 222 women with stage III–IV endometriosis. Following surgery, patients were divided into three groups: placebo, hormone therapy, and a diet enriched with vitamins A, C, E, B6, minerals (calcium, magnesium, selenium, zinc, iron), probiotic cultures, and fish oil. Analysis of symptoms such as dysmenorrhea, dyspareunia, and chronic abdominal pain demonstrated significant pain reduction in all categories in the groups receiving hormone therapy and diet compared to placebo. Moreover, patients in the hormone therapy and diet groups reported a higher quality of life compared to the placebo group [34]. These findings provide evidence that diet plays a crucial role in the development of endometriosis and in the severity of menstrual symptoms.

The analysis of the relationship between diet and menstrual symptoms revealed distinct patterns in the HT and WHT groups. In the WHT group, lower consumption of sweets, red meat, and processed foods was associated with more severe menstrual symptoms such as abdominal pain, weakness, cramps, and irritability. This could result from the avoidance of pro-inflammatory foods that exacerbate symptoms. Women with more severe pain may tend to avoid pro-inflammatory foods, similar to patients with inflammatory bowel disease (IBD), irritable bowel syndrome, or gastroesophageal reflux disease. Studies show that one of the most common strategies for symptom management in IBD is the elimination of so-called “trigger foods” [35]. In fact, 49–90% of IBD patients restrict or completely eliminate specific foods to alleviate symptoms [36]. Patients with non-specific gastrointestinal disorders also frequently eliminate foods perceived as unhealthy [37]. Similarly, women with diagnosed endometriosis who are not undergoing conventional treatment may possess knowledge of dietary recommendations for this condition and consciously eliminate foods that worsen their already severe symptoms. In the study by Ciołek et al., 36.4% of women with endometriosis reported that saturated fats aggravated menstrual symptoms, while 35.4% associated their symptoms with high intake of simple carbohydrates [38].

In the present study, the influence of diet was evident in the HT group, where women receiving hormonal therapy seemed to downplay the role of diet as a therapeutic strategy. These women consumed more salty snacks and sweetened beverages, which were associated with breast tenderness, abdominal pain, and cramps. According to previous reports, frequent consumption of snack foods is associated with a higher risk of moderate to severe dysmenorrhea [39]. Pro-inflammatory dietary patterns, such as high intake of processed meats, sweets, refined grains, and animal fats, were more typical among women with severe dysmenorrhea in a study of 311 healthy Spanish students. In that study, alcohol consumption also influenced pain severity and menstrual cycle length [40]. Similarly, Ciołek et al. found that women with severe dysmenorrhea consumed refined grains, processed meat, and sugar significantly more often than those with moderate menstrual pain [38]. Other research has demonstrated that women with menstrual disorders consume more high-sugar foods and beverages and have inadequate nutrient intake [41]. A preference for sweet, fatty foods was associated with a 2.4-fold higher risk of dysmenorrhea [42]. Numerous studies confirm the negative impact of red and processed meat on inflammation and on the risk of developing endometriosis [43,44,45]. Red meat is particularly associated with elevated estrogen levels in this condition [46].

In this study, regardless of hormonal treatment, higher consumption of wholegrain bread, fish, fruits, and eggs was associated with milder menstrual symptoms such as abdominal pain, cramps, back pain, nausea, and irritability. These food components are known for their anti-inflammatory properties. Ott et al. [47] showed that a diet rich in vegetables, fruits, whole grains, legumes, soy, fatty fish, white meat, and cold-pressed oils—while limiting red meat, sugary drinks, animal fats, and sweets—improved the well-being of women with endometriosis. Reductions in menstrual pain, dyspareunia, discomfort during menstruation, and painful defecation were also observed [47]. Furthermore, a review by Afrin et al. [48] demonstrated that higher intake of Mediterranean diet components, such as vegetables, fruits, and omega-3 fatty acids, may reduce the risk of endometriosis, whereas consumption of trans fats and large amounts of red meat, typical of the Western diet, may increase this risk [48].

The adoption of an anti-endometriosis diet has a beneficial effect on the quality of life of women with diagnosed endometriosis, particularly when adhered to consistently. Its effectiveness is attributed to its anti-inflammatory, antioxidant, and anti-estrogenic properties. Eliminating gluten, dairy, and soy, while increasing vegetable intake, may reduce endometriosis-related pain [49]. These findings are consistent with those of Marziali et al. [50], who demonstrated that adherence to a gluten-free diet among women with endometriosis was associated with significantly better outcomes across all aspects of quality of life [50].

## 5. Limitation

This study has certain limitations. Respondents could fill out the questionnaires subjectively, which could have affected the results. Another limitation is that the division of participants was based solely on hormone therapy status, without creating additional subgroups combining dietary patterns and pharmacological treatment. Such an approach could provide more detailed insight into the interactions between nutrition, hormone therapy and symptom severity. Furthermore, the study was observational and cross-sectional in nature and therefore did not include standard dietary intervention or long-term follow-up, which should be taken into account when interpreting the results. Additionally, it should be noted that severe pain symptoms could have been a criterion for prescribing hormonal drugs, hence the result related to a more severe course of endometriosis in this group. Future studies should include larger populations, use subgroup analyses, and consider longitudinal studies to observe changes over time in order to better investigate the combined effect of hormone therapy and diet on endometriosis symptoms.

## 6. Conclusions

1. In the HT group, women more frequently reported stronger pain and a negative impact of menstruation on work and study compared to the WHT group.
2. Women in the WHT group were more often engaged in professional work, whereas women in the HT group more frequently experienced limitations in daily functioning related to abdominal pain, cramps, and general weakness.
3. In the WHT group, lower consumption of sweets, red meat, and processed products was associated with more severe menstrual symptoms, such as lower abdominal pain, weakness, cramps, and irritability, suggesting that women with more troublesome pain tend to avoid pro-inflammatory foods.
4. In the HT group, higher consumption of salty snacks and sweetened beverages was linked to increased symptoms, including breast tenderness, abdominal pain, and cramps. It is possible that women in this group undergoing treatment do not pay sufficient attention to their diet, perceiving it as a less important factor in alleviating symptoms.
5. In both groups higher consumption of whole-grain bread, fish, fruits, and eggs was associated with milder menstrual symptoms, such as abdominal pain, cramps, back pain, nausea, and irritability.

## Figures and Tables

**Table 1:** Anthropometric characteristics in both groups.

**Group**	**Age [Years]**									
	***N***	$\bar{x}$	**Me**	**Min.**	**Max.**	**Q1**	**Q3**	***SD***	**U**	***p***
**WHT**	100	31.5	32.0	18.0	45.0	28.0	36.0	6.02	4623.50	0.358
**HT**	100	31.4	30.0	24.0	47.0	28.0	34.0	5.35		
**Total**	200	31.5	31.0	18.0	47.0	28.0	35.5	5.68		
**Group**	**Body Weight [kg]**									
	***N***	$\bar{x}$	**Me**	**Min.**	**Max.**	**Q1**	**Q3**	***SD***	**U**	***p***
**WHT**	100	66.4	62.0	40.0	115.0	55.5	74.0	15.64	4784.00	0.599
**HT**	100	64.2	61.5	45.0	105.0	55.0	72.5	11.99		
**Total**	200	65.3	62.0	40.0	115.0	55.0	73.0	13.94		
**Group**	**Height [cm]**									
	***N***	$\bar{x}$	**Me**	**Min.**	**Max.**	**Q1**	**Q3**	***SD***	**U**	***p***
**WHT**	100	167.2	167.5	152.0	178.0	164.0	170.0	5.46	4705.50	0.473
**HT**	100	166.8	167.0	150.0	182.0	163.0	171.0	6.13		
**Total**	200	167.0	167.0	150.0	182.0	164.0	170.0	5.79		
**Group**	**BMI [kg/m^2^]**									
	***N***	$\bar{x}$	**Me**	**Min.**	**Max.**	**Q1**	**Q3**	***SD***	**U**	***p***
**WHT**	100	23.7	22.3	15.6	40.3	20.0	26.0	5.36	4933.00	0.871
**HT**	100	23.0	22.6	16.7	36.3	20.3	25.1	3.79		
**Total**	200	23.4	22.6	15.6	40.3	20.1	25.9	4.64		

*N*—number of observations; $\bar{x}$—mean; Me—Median; Min.—minimum value; Max.—maximum value; Q1—lower quartile; Q3—upper quartile; SD—standard deviation; U—value of the Mann–Whitney U statistic; *p*—test probability value.

**Table 2:** Comparison of menstrual characteristics in the two groups.

**Group**	**Age at First Menstruation [Years]**									
	***N***	$\bar{x}$	**Me**	**Min.**	**Max.**	**Q1**	**Q3**	***SD***	**U**	***p***
**WHT**	100	12.7	13.0	8.0	19.0	12.0	14.0	1.67	4948.50	0.901
**HT**	100	12.6	13.0	8.0	16.0	12.0	13.0	1.40		
**Total**	200	12.6	13.0	8.0	19.0	12.0	13.0	1.54		
**Group**	**Pain Before Menstruation [Days]**									
	***N***	$\bar{x}$	**Me**	**Min.**	**Max.**	**Q1**	**Q3**	***SD***	**U**	***p***
**WHT**	100	3.6	2.0	0.0	30.0	1.0	5.0	4.32	4331.50	0.103
**HT**	100	4.0	3.0	0.0	30.0	2.0	5.0	4.60		
**Total**	200	3.8	2.0	0.0	30.0	1.0	5.0	4.46		
**Group**	**Average Duration of the Menstrual Cycle Length [Days]**									
	***N***	$\bar{x}$	**Me**	**Min.**	**Max.**	**Q1**	**Q3**	***SD***	**U**	***p***
**WHT**	100	29.0	28.0	23.0	38.0	27.0	30.0	2.73	4762.00	0.562
**HT**	100	29.0	28.0	20.0	45.0	28.0	30.0	2.91		
**Total**	200	29.0	28.0	20.0	45.0	28.0	30.0	2.82		
**Group**	**Average Duration of Bleeding [Days]**									
	***N***	$\bar{x}$	**Me**	**Min.**	**Max.**	**Q1**	**Q3**	***SD***	**U**	***p***
**WHT**	100	5.9	6.0	2.0	10.0	5.0	7.0	1.63	4888.50	0.786
**HT**	100	6.0	6.0	1.0	14.0	5.0	7.0	2.04		
**Total**	200	5.9	6.0	1.0	14.0	5.0	7.0	1.84		
**Group**	**Average Pain Level Experienced During Menstruation**									
	***N***	$\bar{x}$	**Me**	**Min.**	**Max.**	**Q1**	**Q3**	***SD***	**U**	***p***
**WHT**	100	6.8	7.0	0.0	10.0	5.5	8.0	2.38	4105.50	**0.029**
**HT**	100	7.5	8.0	0.0	10.0	6.0	9.0	2.00		
**Total**	200	7.1	7.0	0.0	10.0	6.0	9.0	2.22		
**Group**	**Intensity of Bleeding During Menstruation**									
	***N***	$\bar{x}$	**Me**	**Min.**	**Max.**	**Q1**	**Q3**	***SD***	**U**	***p***
**WHT**	100	2.9	3.0	1.0	4.0	2.0	3.5	0.84	4985.00	0.972
**HT**	100	2.9	3.0	1.0	4.0	2.0	4.0	0.86		
**Total**	200	2.9	3.0	1.0	4.0	2.0	4.0	0.85		
**Group**	**Menstrual Discomfort**									
	***N***	$\bar{x}$	**Me**	**Min.**	**Max.**	**Q1**	**Q3**	***SD***	**U**	***p***
**WHT**	100	2.3	3.0	0.0	3.0	2.0	3.0	0.84	4513.00	0.235
**HT**	100	2.5	3.0	0.0	3.0	2.0	3.0	0.70		
**Total**	200	2.4	3.0	0.0	3.0	2.0	3.0	0.78		

WHT—without hormone therapy; HT—hormone therapy; *N*—number of observations; $\bar{x}$—mean; Me—Median; Min.—minimum value; Max.—maximum value; Q1—lower quartile; Q3—upper quartile; SD—standard deviation; U—value of the Mann–Whitney U statistic; *p*—test probability value; intensity of bleeding during menstruation: scanty—1; moderate—2; heavy—3; very heavy—4; menstrual discomfort: none—0; light—1; moderate—2; heavy—3; bold—significance at *p* < 0.05.

**Table 3:** Prevalence of symptoms related to menstruation in both groups.

Symptoms		Group			
		WHT	HT	Total	*p*
**Absence from work/college**	**Yes**	*N* = 32 (32.0%)	*N* = 46 (46.0%)	*N* = 78 (39.0%)	χ^2^ = 4.12 **0.043**
	**No**	*N* = 68 (68.0%)	*N* = 54 (54.0%)	*N* = 122 (61.0%)	
**Lack of concentration**	**Yes**	*N* = 81 (81.0%)	*N* = 86 (86.0%)	*N* = 167 (83.5%)	χ^2^ = 0.91 0.341
	**No**	*N* = 19 (19.0%)	*N* = 14 (14.0%)	*N* = 33 (16.5%)	
**Limitation of** **physical** **activity**	**Yes**	*N* = 91 (91.0%)	*N* = 96 (96.0%)	*N* = 187 (93.5%)	χ^2^ = 1.32 * 0.251
	**No**	*N* = 9 (9.0%)	*N* = 4 (4.0%)	*N* = 13 (6.5%)	
**Adverse impact** **on academic/professional performance**	**Yes**	*N* = 62 (62.0%)	*N* = 79 (79.0%)	*N* = 141 (70.5%)	χ^2^ = 6.95 **0.008**
	**No**	*N* = 38 (38.0%)	*N* = 21 (21.0%)	*N* = 59 (29.5%)	
**Increased** **body temperature**	**Yes**	*N* = 70 (70.0%)	*N* = 58 (58.0%)	*N* = 128 (64.0%)	χ^2^ = 3.13 0.077
	**No**	*N* = 30 (30.0%)	*N* = 42 (42.0%)	*N* = 72 (72.0%)	
**Feeling worse**	**Yes**	*N* = 95 (95.0%)	*N* = 96 (96.0%)	*N* = 191 (95.5%)	χ^2^ = 0.12 0.733
	**No**	*N* = 5 (5.0%)	*N* = 4 (4.0%)	*N* = 9 (9.0%)	

* Chi square with Yates correction; WHT—without hormone therapy; HT—hormone therapy; *N*—number of observations; bold—significance at *p* < 0.05.

**Table 4:** The influence of diet on the symptoms and characteristics of menstruation in the WHT group.

**Absence from Work/College**	**Sugar/Honey**									
	***N***	$\bar{x}$	**Me**	**Min.**	**Max.**	**Q1**	**Q3**	***SD***	**U**	***p***
**Yes**	32	3.2	3.0	1.0	6.0	1.5	5.0	1.68	770.50	**0.019**
**No**	68	2.4	2.0	1.0	6.0	1.0	4.0	1.49		
**Total**	100	2.7	2.0	1.0	6.0	1.0	4.0	1.59		
**Absence from Work/College**	**Cornflakes or Sweet, Flavored Breakfast Cereals**									
	***N***	$\bar{x}$	**Me**	**Min.**	**Max.**	**Q1**	**Q3**	***SD***	**U**	***p***
**Yes**	32	2.2	2.0	1.0	5.0	1.0	3.0	1.15	779.00	**0.023**
**No**	68	1.7	1.0	1.0	5.0	1.0	2.0	1.04		
**Total**	100	1.8	1.0	1.0	5.0	1.0	2.5	1.10		
**Absence from Work/College**	**Sweetened Carbonated Drinks**									
	***N***	$\bar{x}$	**Me**	**Min.**	**Max.**	**Q1**	**Q3**	***SD***	**U**	***p***
**Yes**	32	2.5	2.0	1.0	6.0	1.5	3.0	1.46	804.00	**0.036**
**No**	68	2.0	1.0	1.0	6.0	1.0	3.0	1.46		
**Total**	100	2.2	2.0	1.0	6.0	1.0	3.0	1.47		
**Lack of Concentration**	**Cornflakes or Sweet, Flavored Breakfast Cereals**									
	***N***	$\bar{x}$	**Me**	**Min.**	**Max.**	**Q1**	**Q3**	***SD***	**U**	***p***
**Yes**	81	2.0	2.0	1.0	5.0	1.0	3.0	1.14	474.50	**0.010**
**No**	19	1.3	1.0	1.0	3.0	1.0	1.0	0.65		
**Total**	100	1.8	1.0	1.0	5.0	1.0	2.5	1.10		
**Limiting Physical Activity**	**Fine Groats, White Rice, White Pasta**									
	***N***	$\bar{x}$	**Me**	**Min.**	**Max.**	**Q1**	**Q3**	***SD***	**U**	***p***
**Yes**	91	3.0	3.0	1.0	5.0	2.0	4.0	1.13	240.50	**0.042**
**No**	9	2.1	2.0	1.0	4.0	1.0	3.0	1.17		
**Total**	100	2.9	3.0	1.0	5.0	2.0	4.0	1.15		
**Limiting Physical Activity**	**Juices, Nectars, Drinks**									
	***N***	$\bar{x}$	**Me**	**Min.**	**Max.**	**Q1**	**Q3**	***SD***	**U**	***p***
**Yes**	91	2.7	2.0	1.0	6.0	1.0	4.0	1.48	242.50	**0.045**
**No**	9	1.7	1.0	1.0	4.0	1.0	2.0	1.12		
**Total**	100	2.6	2.0	1.0	6.0	1.0	4.0	1.48		
**Adverse Impact on Academic/Work Performance**	**Sugar/Honey**									
	***N***	$\bar{x}$	**Me**	**Min.**	**Max.**	**Q1**	**Q3**	***SD***	**U**	***p***
**Yes**	62	2.9	3.0	1.0	6.0	1.0	4.0	1.58	895.00	**0.045**
**No**	38	2.2	1.5	1.0	6.0	1.0	3.0	1.55		
**Total**	100	2.7	2.0	1.0	6.0	1.0	4.0	1.59		
**Adverse Impact on Academic/Work Performance**	**Cornflakes or Sweet, Flavored Breakfast Cereals**									
	***N***	$\bar{x}$	**Me**	**Min.**	**Max.**	**Q1**	**Q3**	***SD***	**U**	***p***
**Yes**	62	2.1	2.0	1.0	5.0	1.0	3.0	1.16	790.00	**0.006**
**No**	38	1.4	1.0	1.0	5.0	1.0	2.0	0.86		
**Total**	100	1.8	1.0	1.0	5.0	1.0	2.5	1.10		
**Adverse Impact on Academic/Work Performance**	**Fine Groats, White Rice, White Pasta**									
	***N***	$\bar{x}$	**Me**	**Min.**	**Max.**	**Q1**	**Q3**	***SD***	**U**	***p***
**Yes**	62	3.2	3.0	1.0	5.0	3.0	4.0	1.11	808.50	**0.009**
**No**	38	2.6	3.0	1.0	5.0	2.0	3.0	1.13		
**Total**	100	2.9	3.0	1.0	5.0	2.0	4.0	1.15		
**Adverse Impact on Academic/Work Performance**	**Vegetable Fats**									
	***N***	$\bar{x}$	**Me**	**Min.**	**Max.**	**Q1**	**Q3**	***SD***	**U**	***p***
**Yes**	62	3.9	4.0	1.0	6.0	3.0	5.0	1.20	882.00	**0.036**
**No**	38	3.3	3.0	1.0	6.0	2.0	4.0	1.37		
**Total**	100	3.6	4.0	1.0	6.0	3.0	5.0	1.29		
**Adverse Impact on Academic/Work Performance**	**Juices, Nectars, Drinks**									
	***N***	$\bar{x}$	**Me**	**Min.**	**Max.**	**Q1**	**Q3**	***SD***	**U**	***p***
**Yes**	62	2.9	3.0	1.0	6.0	2.0	4.0	1.52	840.00	**0.017**
**No**	38	2.1	2.0	1.0	5.0	1.0	3.0	1.29		
**Total**	100	2.6	2.0	1.0	6.0	1.0	4.0	1.48		
**Adverse Impact on Academic/Work Performance**	**Fast Foods**									
	***N***	$\bar{x}$	**Me**	**Min.**	**Max.**	**Q1**	**Q3**	***SD***	**U**	***p***
**Yes**	62	2.6	2.0	1.0	5.0	2.0	3.0	1.15	883.50	**0.037**
**No**	38	2.1	2.0	1.0	4.0	1.0	3.0	0.90		
**Total**	100	2.4	2.0	1.0	5.0	2.0	3.0	1.09		
**Increased Temperature Ciała**	**Cornflakes or Sweet, Flavored Breakfast Cereals**									
	***N***	$\bar{x}$	**Me**	**Min.**	**Max.**	**Q1**	**Q3**	***SD***	**U**	***p***
**Yes**	30	2.2	2.0	1.0	5.0	1.0	3.0	1.15	770.50	**0.036**
**No**	70	1.7	1.0	1.0	5.0	1.0	2.0	1.05		
**Total**	100	1.8	1.0	1.0	5.0	1.0	2.5	1.10		
**Feeling Worse**	**Sugar/Honey**									
	***N***	$\bar{x}$	**Me**	**Min.**	**Max.**	**Q1**	**Q3**	***SD***	**U**	***p***
**Yes**	95	2.7	3.0	1.0	6.0	1.0	4.0	1.59	82.50	**0.015**
**No**	5	1.0	1.0	1.0	1.0	1.0	1.0	0.00		
**Total**	100	2.7	2.0	1.0	6.0	1.0	4.0	1.59		

*N*—number of observations; $\bar{x}$—mean; Me—Median; Min.—minimum value; Max.—maximum value; Q1—lower quartile; Q3—upper quartile; SD—standard deviation; U—value of the Mann–Whitney U statistic; *p*—test probability value; bold—significance at *p* < 0.05.

**Table 5:** Effect of diet on menstrual symptoms and characteristics in the HT group.

**Absence from Work/College**	**Nuts**									
	***N***	$\bar{x}$	**Me**	**Min.**	**Max.**	**Q1**	**Q3**	***SD***	**U**	***p***
**Yes**	46	4.0	4.0	1.0	6.0	3.0	5.0	1.35	916.00	**0.024**
**No**	54	3.4	3.5	1.0	6.0	2.0	4.0	1.36		
**Total**	100	3.7	4.0	1.0	6.0	3.0	5.0	1.39		
**Absence from Work/College**	**Vegetables**									
	***N***	$\bar{x}$	**Me**	**Min.**	**Max.**	**Q1**	**Q3**	***SD***	**U**	***p***
**Yes**	46	5.4	6.0	3.0	6.0	5.0	6.0	0.80	817.00	**0.003**
**No**	54	4.8	5.0	2.0	6.0	4.0	6.0	0.97		
**Total**	100	5.1	5.0	2.0	6.0	4.0	6.0	0.94		
**Lack of Concentration**	**Cheese, Processed Cheese, Blue Cheese**									
	***N***	$\bar{x}$	**Me**	**Min.**	**Max.**	**Q1**	**Q3**	***SD***	**U**	***p***
**Yes**	86	3.3	3.0	1.0	6.0	2.0	4.0	1.45	401.50	**0.047**
**No**	14	4.1	4.0	3.0	5.0	4.0	4.0	0.62		
**Total**	100	3.4	4.0	1.0	6.0	2.5	4.0	1.39		
**Lack of Concentration**	**Oat, Barley and Rye Flakes**									
	***N***	$\bar{x}$	**Me**	**Min.**	**Max.**	**Q1**	**Q3**	***SD***	**U**	***p***
**Yes**	86	3.4	4.0	1.0	6.0	2.0	4.0	1.52	386.50	**0.033**
**No**	14	2.4	2.0	1.0	5.0	1.0	4.0	1.39		
**Total**	100	3.2	3.0	1.0	6.0	2.0	4.0	1.53		
**Limiting Physical Activity**	**White Bread**									
	***N***	$\bar{x}$	**Me**	**Min.**	**Max.**	**Q1**	**Q3**	***SD***	**U**	***p***
**Yes**	96	3.6	4.0	1.0	6.0	2.5	5.0	1.50	55.50	**0.017**
**No**	4	5.5	6.0	4.0	6.0	5.0	6.0	1.00		
**Total**	100	3.7	4.0	1.0	6.0	3.0	5.0	1.53		
**Limiting Physical Activity**	**Oat, Barley and Rye Flakes**									
	***N***	$\bar{x}$	**Me**	**Min.**	**Max.**	**Q1**	**Q3**	***SD***	**U**	***p***
**Yes**	96	3.3	4.0	1.0	6.0	2.0	4.0	1.52	68.00	**0.030**
**No**	4	1.5	1.5	1.0	2.0	1.0	2.0	0.58		
**Total**	100	3.2	3.0	1.0	6.0	2.0	4.0	1.53		
**Limiting Physical Activity**	**Buckwheat, Barley Groats, Brown Rice, Wholemeal Pasta**									
	***N***	$\bar{x}$	**Me**	**Min.**	**Max.**	**Q1**	**Q3**	***SD***	**U**	***p***
**Yes**	96	3.4	3.0	1.0	6.0	3.0	4.0	1.17	43.50	**0.009**
**No**	4	1.5	1.0	1.0	3.0	1.0	2.0	1.00		
**Total**	100	3.3	3.0	1.0	6.0	2.5	4.0	1.22		
**Limiting Physical Activity**	**Fine Groats, White Rice, White Pasta**									
	***N***	$\bar{x}$	**Me**	**Min.**	**Max.**	**Q1**	**Q3**	***SD***	**U**	***p***
**Yes**	96	3.4	3.0	1.0	6.0	3.0	4.0	1.16	63.00	**0.024**
**No**	4	2.0	2.0	1.0	3.0	1.5	2.5	0.82		
**Total**	100	3.4	3.0	1.0	6.0	3.0	4.0	1.18		
**Adverse Impact on Academic/Work Performance**	**Animal Fats**									
	***N***	$\bar{x}$	**Me**	**Min.**	**Max.**	**Q1**	**Q3**	***SD***	**U**	***p***
**Yes**	79	2.7	2.0	1.0	6.0	1.0	4.0	1.59	564.00	**0.025**
**No**	21	3.7	4.0	1.0	6.0	2.0	5.0	1.71		
**Total**	100	2.9	3.0	1.0	6.0	1.0	4.0	1.66		

*N*—number of observations; $\bar{x}$—mean; Me—Median; Min.—minimum value; Max.—maximum value; Q1—lower quartile; Q3—upper quartile; SD—standard deviation; U—value of the Mann–Whitney U statistic; *p*—test probability value; bold—significance at *p* < 0.05.

## Data Availability

The original contributions presented in this study are included in the article/Supplementary Material. Further inquiries can be directed to the corresponding author.

## Supplementary Materials

The following supporting information can be downloaded at https://www.mdpi.com/article/10.3390/nu17172889/s1, Supplementary File S1: Anonymous survey, Supplementary Table S1: Assessment of the relationship between diet and menstrual symptoms in the study and control groups.

## Abbreviations

The following abbreviations are used in this manuscript:

IBD	inflammatory bowel disease
WHT	Women with endometriosis without hormone therapy
WT	Women with hormone therapy
VAS	Visual Analog Scale
