# Supplementary material for: The Impact of Nutrition on Endometriosis Complaints in Patients Using and Not Using Hormone Therapy
Source: Nutrients. 2025 Sep 6;17(17):2889. doi: 10.3390/nu17172889 (PMC12430643; doi:10.3390/nu17172889)
Supplement: Supplementary file 1 [file nutrients-17-02889-s001.zip › nutrients-3814655-supplementary.pdf]

Supplementary Files S1 - Anonymous Survey (English Translation)

Students' Scientific Circle of Dietetics – Food Safety, Institute of Health Sciences, College of Medical Sciences, University of Rzeszów invites you to participate in a survey study aimed at assessing the impact of dietary habits on the intensity of pain and symptoms associated with menstruation in women struggling with endometriosis.

The questionnaire is ANONYMOUS, voluntary, and intended exclusively for scientific purposes.

Each respondent may withdraw consent for the processing of collected data at any time during the study, in accordance with applicable Polish law (Act of May 10, 2018 on the protection of personal data – Journal of Laws 2018, item 1000), and may withdraw participation at any time without providing a reason.

We sincerely thank you for taking part in this survey!

Demographic Questions

1. **Age ……………… years Body weight …………... kg Height ………………….. cm**
2. **Place of residence:**
   - Village
   - City up to 100,000 inhabitants
   - City over 100,000 inhabitants
3. **Education:**
   - Primary/Lower secondary
   - Vocational
   - Secondary
   - Higher

Medical Background

4. **Age at first menstruation:** …………… years
5. **Days before menstruation when pain appears:** …………… day(s)
6. **Average length of menstrual cycle:** …………… days
7. **Average duration of bleeding:** …………… days
8. **Bleeding intensity:**
   - Light
   - Moderate
   - Heavy
   - Very heavy
9. **Regular menstruation in last 6 months?**
   - Yes
   - No
10. **Burden of menstruation:**
    - None
    - Mild
    - Moderate
    - Severe
11. **Average pain level (0–10): □0 □1 □2 □3 □4 □5 □6 □7 □8 □9 □10**

Menstruation-Related Symptoms

12. **Do you experience the following?**

	**YES**	**NO**
- Absence from work/university		
- Lack of concentration		
- Limitation of physical activity		
- Negative impact on academic/work performance		
- Elevated body temperature		
- Worse well-being compared to premenstrual days		

13. **Duration of symptoms:**

	**None**	**- First 1 day**	**- 2–3 days**	**- 4–6 days**	**- More than 6 days**
- Lower abdominal pain					
- Back pain					
- Headache					
- Weakness					
- Abdominal cramps					
-Irritability/nervousness					
- Breast tenderness					
- Nausea/vomiting					

Fertility

14. **Do you have children?**
    - Yes
    - No
15. **Have you experienced infertility problems related to endometriosis?**
    - Yes
    - No
    - I don’t have children
16. **How long have you been trying to have children?**
    - Up to 6 months

- 6 months – 1 year
- 1–3 years
- 1–5 years
- Over 5 years
- I don't have children

Dietary Habits (last 6 months)
The following questions were developed based on the FFQ-6 questionnaire, the purpose of which is to collect information about dietary habits over the LAST 6 MONTHS.

17. Jak często spożywa Pani?

	**Several times a day**	**Daily**	**Several times a week**	**Several times a month**	**Once a month or less**	**Never or almost never**
Cakes, cookies,						
Candy bars, chocolates, chocolates, ice cream, puddings, etc.						
Sugar or honey to sweeten drinks and meals						
Salty snacks, e.g., chips, salted crisps, crackers, breadsticks						
Natural milk						
Natural sour milk products (buttermilk, kefir, yogurt)						
Cheese (cottage cheese, processed cheese, blue cheese)						
Eggs and egg dishes						
White bread, e.g., wheat bread, rolls, toasted bread						
Dark bread, e.g., whole wheat bread,						

graham bread, whole wheat rolls						
Pastry goods, e.g., doughnuts, buns, croissants						
Corn or sweet breakfast cereals, flavored						
Oat flakes, barley flakes, rye flakes						
Buckwheat groats, barley groats, brown rice, whole wheat pasta						
Small grain groats, semolina Pearl or Krakowska barley, white rice, white pasta						
Potatoes						
Vegetable fats: oils, margarine						
Animal fats: butter, lard, bacon						
Fresh fruit						
Dried fruit						
Nuts: walnuts, hazelnuts, peanuts, and others, sunflower and pumpkin seeds						
Vegetables of all kinds						
Dried legumes - beans, peas, chickpeas						

Red meat (pork, beef, lamb)						
Poultry - white meat						
Fish						
Cold cuts, hot dogs, sausages, kabanos sausages, etc.						
Fruit juices, nectars, fruit drinks						
Sweetened carbonated drinks (cola, Pepsi, Mirinda, Fanta, orange soda)						
Alcohol						
Fast food						
Organic products						
Alcohol						
Cigarettes						

Supplements and Diet

18. Do you take dietary supplements? If yes, which?
- Yes: …………………
- No

19. Are you on a diet? If yes, which?
- Yes: …………………
- No

20. How are your meals prepared?

- Boiled
- Steamed
- Fried
- Baked
- Smoked

Physical Activity

21. Do you engage in physical activity?
- Yes
- No

22. Frequency:
- Less than once a month
- Once a month
- Several times a month
- Once a week
- Several times a week
- Daily

23. Average training duration:
- Less than 30 minutes
- Less than 1 hour
- More than 1 hour
- About 2 hours

Lifestyle and Quality of Life

24. Do you believe diet has an impact on endometriosis symptoms?
- Yes
- No

25. How do you rate your quality of life? (0 – very poor, 10 – very good)

□0 □1 □2 □3 □4 □5 □6 □7 □8 □9 □10

Supplementary Table S1. Assessment of the relationship between diet and menstrual symptoms in the study and control groups.

*R	1	2	3	4	5	6	7	8
**Study group**								
**Cakes, cookies**	-0.12	-0.08	-0.04	-0.12	**-0.20**	-0.15	-0.14	0.00
**Bars, chocolates, chocolates**	-0.09	-0.07	-0.04	-0.06	-0.18	-0.03	-0.07	0.03
**Sugar/honey**	0.01	0.00	0.03	0.08	-0.01	0.00	-0.07	0.13
**Salty snacks**	-0.03	0.01	0.02	-0.05	-0.13	0.04	0.09	**0.22**
**Milk**	0.11	**0.28**	**0.22**	0.16	0.02	0.12	0.17	0.02
**Natural sour milk products**	0.01	0.12	0.10	0.00	0.02	0.07	0.09	-0.02
**Flavored, sweetened sour milk products**	0.05	0.05	0.05	0.08	0.13	0.09	0.05	-0.04
**Cottage cheese**	0.06	0.08	0.07	0.09	0.00	0.10	0.07	-0.10
**Cheese, processed cheese, blue cheese**	-0.01	-0.06	0.05	0.06	0.01	0.05	0.00	0.08
**Eggs**	0.07	0.15	0.06	0.10	0.06	0.13	0.03	0.12
**White bread**	-0.06	-0.09	-0.08	0.05	-0.06	0.00	0.05	0.03
**Whole wheat bread**	-0.10	0.01	-0.07	-0.17	-0.07	-0.08	-0.03	-0.13
**Confectionery bread**	-0.02	0.07	0.04	-0.09	-0.15	-0.19	-0.06	0.02
**Cornflakes or sweet, flavored breakfast cereals**	0.11	0.19	**0.20**	**0.20**	0.15	0.13	0.04	**0.21**
**Oat, barley and rye flakes**	0.07	0.16	0.02	-0.01	0.02	0.10	0.03	0.01
**Buckwheat, barley groats, brown rice, wholemeal pasta**	0.14	0.17	0.11	0.18	0.18	**0.21**	0.04	0.03

**Fine groats, white rice, white pasta**	0.09	0.05	-0.01	0.12	0.11	0.19	0.10	0.06
**Potatoes**	0.09	0.12	-0.06	0.14	0.07	0.01	0.10	0.10
**Vegetable fats**	**0.29**	0.17	0.05	**0.25**	**0.21**	**0.22**	0.01	0.14
**Animal fats**	-0.10	-0.03	-0.04	-0.07	-0.08	-0.06	-0.17	0.08
**Fresh fruits**	0.05	0.03	-0.03	0.05	0.06	0.11	0.04	0.10
**Dry fruits**	-0.18	-0.03	0.05	-0.13	-0.15	-0.13	-0.17	0.12
**Nuts**	-0.03	0.00	-0.07	-0.11	-0.04	0.03	-0.15	-0.03
**Vegetables**	0.07	0.05	-0.12	-0.01	0.08	0.07	0.06	-0.04
**Legume seeds**	0.03	0.05	-0.08	-0.12	0.00	-0.09	-0.04	0.09
**Red meat**	**-0.30**	-0.13	-0.11	**-0.20**	**-0.26**	-0.11	-0.15	0.06
**Poultry**	-0.02	-0.03	-0.04	-0.01	0.01	0.13	-0.03	-0.03
**Fish**	-0.02	0.05	0.02	0.00	0.08	0.04	0.12	0.07
**Cold cuts, frankfurters, sausages**	**-0.20**	-0.06	0.01	-0.08	-0.18	-0.10	-0.06	0.15
**Juices, nectars, drinks**	-0.10	-0.05	-0.08	-0.02	-0.05	-0.12	-0.11	0.13
**Sweetened carbonated drinks**	0.06	0.04	0.03	0.06	0.02	-0.04	-0.09	0.05
**Alcohol**	-0.02	0.05	-0.02	0.01	0.00	-0.01	0.01	0.09
**Fast foods**	0.06	0.13	0.09	0.05	-0.06	0.06	-0.03	0.13
**Control group**								
**Cakes, cookies**	-0.08	-0.14	-0.02	0.08	-0.02	-0.05	-0.06	-0.10
**Bars, chocolates, chocolates**	-0.12	-0.09	-0.02	0.06	-0.06	0.07	-0.02	-0.09
**Sugar/honey**	0.07	0.05	0.09	0.12	0.06	0.06	0.06	-0.05

**Salty snacks**	-0.05	-0.01	-0.01	0.06	-0.05	0.05	**0.23**	-0.11
**Milk**	0.02	0.09	0.10	0.06	0.03	0.14	0.12	0.11
**Natural sour milk products**	-0.04	0.04	-0.02	-0.03	-0.09	0.00	0.01	0.09
**Flavored, sweetened sour milk products**	-0.03	-0.06	0.03	0.02	-0.05	0.03	-0.04	-0.07
**Cottage cheese**	-0.05	-0.04	0.02	0.07	-0.09	0.03	0.06	-0.05
**Cheese, processed cheese, blue cheese**	0.07	0.07	0.11	0.10	0.07	0.11	0.02	0.00
**Eggs**	-0.07	-0.07	-0.13	-0.15	-0.16	**-0.21**	-0.16	-0.13
**White bread**	0.01	0.02	0.05	0.05	0.03	0.08	0.11	**0.21**
**Whole wheat bread**	-0.16	**-0.25**	-0.16	**-0.22**	**-0.25**	-0.17	-0.10	-0.09
**Confectionery bread**	-0.05	-0.16	0.00	-0.01	-0.13	-0.04	-0.06	-0.03
**Cornflakes or sweet, flavored breakfast cereals**	-0.17	**-0.22**	-0.12	0.04	-0.18	-0.08	0.00	-0.10
**Oat, barley and rye flakes**	**-0.27**	-0.18	0.04	-0.07	**-0.22**	-0.14	-0.09	0.02
**Buckwheat, barley groats, brown rice, wholemeal pasta**	-0.19	-0.08	-0.10	-0.13	-0.16	-0.14	-0.08	-0.07
**Fine groats, white rice, white pasta**	-0.06	-0.05	0.12	0.05	-0.04	0.06	0.07	0.09
**Potatoes**	-0.08	0.00	-0.04	-0.03	-0.03	-0.05	-0.10	0.00
**Vegetable fats**	-0.06	0.02	-0.08	-0.03	0.00	-0.02	-0.02	-0.02
**Animal fats**	0.12	**0.23**	-0.01	0.19	**0.20**	0.17	0.10	0.03
**Fresh fruits**	-0.17	-0.08	0.04	-0.15	-0.12	-0.19	**-0.20**	0.11
**Dry fruits**	-0.09	-0.10	0.13	-0.03	-0.12	-0.09	-0.06	0.03
**Nuts**	-0.05	-0.01	0.12	-0.02	-0.06	-0.06	-0.11	0.03

**Vegetables**	0.05	0.08	-0.01	-0.10	0.00	-0.07	-0.13	0.18
**Legume seeds**	-0.08	-0.11	0.10	0.02	-0.10	-0.01	-0.05	0.11
**Red meat**	-0.01	-0.12	0.01	0.03	-0.02	0.00	-0.01	-0.08
**Poultry**	-0.14	-0.13	-0.06	-0.09	-0.11	-0.18	**-0.22**	-0.12
**Fish**	-0.16	**-0.22**	-0.08	**-0.21**	**-0.26**	**-0.20**	**-0.32**	-0.09
**Cold cuts, frankfurters, sausages**	-0.05	-0.05	0.00	0.02	-0.05	0.00	0.00	-0.16
**Juices, nectars, drinks**	-0.09	-0.12	0.00	0.10	-0.08	0.05	0.17	-0.01
**Sweetened carbonated drinks**	0.00	-0.03	-0.01	0.15	0.00	0.12	**0.20**	0.10
**Alcohol**	0.07	0.07	-0.04	0.10	0.04	0.11	0.12	0.13
**Fast foods**	0.03	-0.04	-0.07	-0.01	-0.01	0.08	0.07	0.10

*R – Spearman's rank correlation coefficient values /statistically significant correlations at the level of $p<0.05$ are marked in bold; 1 – lower abdominal pain; 2 – backache; 3 – headache; 4 – weakness; 5 – cramps felt in the lower abdomen; 6 – irritability/nervousness; 7 – breast tenderness; 8 – nausea/vomiting
